# Development and evaluation of a patient centered cardiovascular health education program for insured patients in rural Nigeria (QUICK - II)

**DOI:** 10.1186/1471-2458-11-171

**Published:** 2011-03-21

**Authors:** Aina Olufemi Odusola, Marleen Hendriks, Constance Schultsz, Karien Stronks, Joep Lange, Akin Osibogun, Tanimola Akande, Shade Alli, Peju Adenusi, Kayode Agbede, Joke Haafkens

**Affiliations:** 1 Dept of Global Health, Academic Medical Center, University of Amsterdam, Pietersbergweg 17, Amsterdam, 1105 BM, The Netherlands; 2 PharmAccess Foundation, 1c Raymond Njoku Street, S.W. Ikoyi, Lagos, Nigeria; 3 Dept of Neurology, Academic Medical Center, University of Amsterdam, Meibergdreef 9, Amsterdam, 1105 AZ, The Netherlands; 4 Oxford University Clinical Research Unit, Hospital for Tropical Diseases, 190 Ben Ham Tu, Ho Chi Min City, District 5, Vietnam; 5 Dept of Public Health, Academic Medical Center, University of Amsterdam, Meibergdreef 9, Amsterdam, 1105 AZ, The Netherlands; 6 Dept of Community Health, Lagos University Teaching Hospital, P.M.B.12003, Idi-Araba, Surulere, Lagos, Nigeria; 7 Dept of Epidemiology and Community Health, University of Ilorin Teaching Hospital, P.M.B. 1459, Ilorin, postal code 240001, Nigeria; 8 Dept of Cardiology, Lagoon Hospitals, 8 Marine Road, Apapa, Lagos, Nigeria; 9 Hygeia Nigeria Ltd, 13B Idejo Street, Victoria Island, Lagos, Nigeria; 10 Ogo Oluwa Hospital, 64/65 Ahmadu Bello Way, Bacita, Kwara State, Nigeria; 11 Dept of General Practice, Academic Medical Center, University of Amsterdam, Meibergdreef 9, Amsterdam, 1105 AZ, The Netherlands

## Abstract

**Background:**

In Sub Saharan Africa, the incidence of hypertension and other modifiable cardiovascular risk factors is growing rapidly. Poor adherence to prescribed prevention and treatment regimens by patients can compromise treatment outcomes. Patient-centered cardiovascular health education is likely to improve shortcomings in adherence. This paper describes a study that aims to develop a cardiovascular health education program for patients participating in a subsidized insurance plan in Nigeria and to evaluate the applicability and effectiveness in patients at increased risk for cardiovascular disease.

**Methods/Design:**

*Design: *The study has two parts. Part *1 *will develop a cardiovascular health education program, using qualitative interviews with stakeholders. Part *2 *will evaluate the effectiveness of the program in patients, using a prospective (pre-post) observational design.

*Setting: *A rural primary health center in Kwara State, Nigeria.

*Population: *For part 1: 40 patients, 10 healthcare professionals, and 5 insurance managers. For part 2: 150 patients with uncontrolled hypertension or other cardiovascular risk factors after one year of treatment.

*Intervention: *Part *2*: patient-centered cardiovascular health education program.

*Measurements: *Part 1: Semi-structured interviews to identify stakeholder perspectives. Part 2: Pre- and post-intervention assessments including patients' demographic and socioeconomic data, blood pressure, body mass index and self-reporting measures on medication adherence and perception of care. Feasibility of the intervention will be measured using process data.

*Outcomes: *For program development (part 1): overview of healthcare professionals' perceptions on barriers and facilitators to care, protocol for patient education, and protocol implementation plan.

For program evaluation (part 2): changes in patients' scores on adherence to medication and life style changes, blood pressure, and other physiological and self-reporting measures at six months past baseline.

*Analysis: *Part 1: content analytic technique utilizing MAXQDA software. Part 2: univariate and multilevel analysis to assess outcomes of intervention.

**Discussion:**

Diligent implementation of patient-centered education should enhance adherence to cardiovascular disease prevention and management programs in low income countries.

**Trial Registration:**

ISRCTN47894401

## Background

Sub Saharan African (SSA) countries are currently experiencing a rapid increase in the incidence of cardiovascular diseases (CVD) [1,2]. Hypertension is an important risk factor for CVD. Poor adherence to prescribed medication regimens or lifestyle advices can severely compromise the effectiveness of CVD prevention and treatment [3]. For that reason, the World Health Organization (WHO) emphasizes in a recent report that any attempt to improve cardiovascular care should also address the issue on non-adherence [3]. The views and beliefs of patients regarding disease conditions and treatment may differ from medical perceptions, and it is well documented that patients do have significant roles to play in adherence to medications and lifestyle measures [4-6]. Evidence to date indicates that patient education is one of the most successful interventions to improve adherence and patient self-management of chronic diseases, especially if the education addresses patients' beliefs and concerns about the condition and treatment, identifies social cultural and individual barriers to adherence and enhances patients' confidence in their ability to overcome those barriers [7].

In this paper, we describe the design of a study that has the aim to develop and test a program for cardiovascular health education for patients who are enrolled in a subsidized, community-based health insurance program in Nigeria and are at an increased risk of developing CVD. This study is part of the project "QUality Improvement for Cardiovascular care Kwara (QUICK)". The project will be evaluated by two studies: QUICK-I and QUICK-II. This paper describes the design of the QUICK-II study that focuses on patient education. A detailed description of the insurance program and the QUICK-I study can be found elsewhere [8].

### Aim of the study

The World Health Organization/International Society of Hypertension (WHO/ISH) guidelines recommend patient education as part of CVD prevention care [9], but they do not provide clear recommendations on how this education should be delivered and tailored to the specific target groups in the region. We hypothesize that tailored patient education will improve adherence to cardiovascular care among patients.

For that reason, the main aims of QUality Improvement Cardiovascular care Kwara II (QUICK - II) are:

1. To develop and implement a targeted cardiovascular health education program (CHEP) for patients participating in the Hygeia Community Health Plan (HCHP)

2. To evaluate the newly developed program with respect to its applicability and effectiveness in patients at an increased risk for CVD

## Methods

### Project design

#### QUICK - II consist of two consecutive parts

In *part 1 *of the study, we will develop a stakeholder based Cardiovascular Health Education Program (CHEP) that is to be used to educate patients at risk of CVD who are enrolled in a private health insurance plan in rural Nigeria. To develop CHEP, we will use the following step-wise approach: (i) open qualitative interviews will be conducted with key stakeholders in CVD care to explore their perceptions on CVD, CVD risk factor management and CVD prevention and care. These stakeholders include patients at risks for CVD, healthcare providers (HCP), and health insurance managers of HCHP; (ii) on the basis of the outcomes of interviews with patients, CHEP will be developed; (iii) supportive strategies needed to implement CHEP successfully will be identified on the basis of the outcomes of the interviews with HCP and health insurance managers.

In *Part 2 *of the study we aim to evaluate the effect of CHEP through a prospective hospital-based study, using a pre-post intervention design. Measurements will be conducted in a subset of patients included in QUICK - I: those who have uncontrolled hypertension or other CVD risk factors, or are non adherent to medication after 12 months of treatment.

In addition, case file data will be reviewed and interviews with health care professionals in the participating clinic will be held and analyzed to evaluate the feasibility of the application of CHEP in practice.

### Setting

Ogo Oluwa Hospital (OOH) in Bacita (Kwara State). A detailed description of this setting can be found in the accompanying paper describing the QUICK-I study design [8].

Because part 1 and part 2 of this study have different designs, we will describe the study procedures in different sections.

## Part 1: Development of Chep

### Study Population

The study population for Part 1 will consist of four groups: *Group 1 *- A purposeful sample of 20 patients with 'controlled hypertension' equally distributed by gender (50% male and female), and age (18-35 yrs, 36-55 yrs, 56 yrs and over) who were included in the QUICK - I study; *Group 2 *- A purposeful sample of 20 patients with 'uncontrolled hypertension' equally distributed by gender (50% male and female), and age (18-35 yrs, 36-55 yrs, 56 yrs and over) who were included in QUICK - I study; *Group 3 *- Eight to ten healthcare professionals treating patients with hypertension (HTN), diabetes mellitus (DM) or CVD at OOH; and *Group 4 *- Five to ten managers and doctors of the Health Maintenance Organization (HMO) Hygeia.

### Sample size and Recruitment

#### Sample size

For qualitative interviews, data saturation is a criterion for calculation of the sample size. In general, about 20 interviews are required until saturation is reached and no new information on the major themes is collected [10,11]. For that reason we decided to interview 40 patients (20 with controlled and 20 with uncontrolled hypertension). The number of interviews for HCP is less because the number of health care professionals working in the region is limited. The same accounts for health insurance managers and doctors of Hygeia HMO.

#### Patient recruitment

The patients in Groups 1 and 2 will be recruited among participants in the QUICK - I study in the first month after their inclusion. Health care professionals (Group 3); and managers and doctors working for Hygeia HMO (Group 4) will be included in the first 3 months of QUICK - I. Eligible respondents will be adequately informed of the objectives of the qualitative study. Permission for the interview and written or tape-recorded informed consents will be taken.

### Outcomes

The outcomes envisaged for part 1 include: (i) an overview of patients' perceptions on CVD, CVD risk factors and inhibiting or facilitating factors for CVD prevention and care; (ii) an overview of perceptions of HCP, and health insurance managers and doctors of Hygeia HMO on barriers and facilitators to implementation of CVD prevention and care; (iii) a protocol for CHEP; and (iv) a plan of supportive strategies for implementation of CHEP, including training for HCP.

### Materials and measurements

In part one, patients allocated to Groups 1 and 2 will be interviewed using a semi-structured questionnaire that is based on a topic list used in a similar study by Beune et al [12], but modified to suit the Nigerian setting. Healthcare professionals from Groups 3 and 4 will similarly be interviewed using semi structured questionnaires based on topic lists specifically designed to address the particular concerns of these groups. A researcher who speaks English and Yoruba fluently, assisted by interpreters in Nupe, the other dominant local language, will conduct interviews. CHEP will be developed based on a systematic review of literature on similar past patient education programs, and analysis of the interview data.

### Data management and analysis

#### Data entry and cleaning

The researcher will transcribe the semi-structured interviews conducted in part 1 and check unclear passages with respondents, if needed.

#### Data analysis

The transcribed qualitative interview data will be analyzed using content analytical techniques: fragments containing the respondents' ideas about major themes, for example inhibitors or facilitators of cardiovascular diseases care, will be identified from each interview and coded. Similar codes will be assigned to related statements, resulting in a code list for each interview. Code lists will be compared to identify common and unique themes, leading to a thematic matrix for each group of respondents. Similarities, variations and patterns between groups will be compared, using these matrices.

Data entry and analysis will be supported by MAXQDA software http://www.maxqda.com. MAXQDA facilitates data management, the assignment of labels, codes and themes to text fragments and the generation of thematic matrices containing these elements. In the past, MAXQDA software has been successfully used in similar studies [13].

## Part 2: Evaluation of Chep

### Study population

To be included in the evaluation study of the effect of CHEP (part 2), patients have to meet the following inclusion criteria. They must: (i) be enrolled in HCHP; (ii) be registered as a patient in OOH; (iii) be included in the QUICK - I study for at least 12 months; (iv) have a diagnosis of hypertension; (v) have uncontrolled BP (≥140 mmHg systolic or ≥90 mmHg diastolic) or be non adherent to prescribed medication or any other recommended life style changes according to their score on the Morisky scale [14]; and (vi) be ≥18 years of age. Patients who meet these inclusion criteria will be excluded if their treating health care professional judges them unfit for participation (e.g. due to co morbidity) or if they are not capable or unwilling to give informed consent. Female patients who are pregnant or lactating will be excluded from the entire study because the etiology, prognosis and treatment regimes of gestational diabetes and pregnancy-induced hypertension are different compared to hypertension and diabetes in non-pregnant women.

To be included in the qualitative interviews for the feasibility analysis, healthcare professionals must: (i) be a HCP in OOH who participated in the implementation of CHEP, or be managers and doctors of HCHP; and (ii) give informed consent.

Table 1 shows an overview of the inclusion and exclusion criteria for CHEP study. For an overview of the inclusion and exclusion criteria of QUICK - I, see the article of the QUICK - I study design [8].

**Table 1:** Inclusion and exclusion criteria for CHEP study.

Inclusion Criteria	Exclusion Criteria
Patients:	Patients:
• Enrollment in HCHP	• Unwillingness to give informed consent
• Registered and accessing care in OOH	• Unfit for participation (e.g. due to co morbidity)
• Inclusion in QUICK - I for at least 12 months	• Pregnant or lactating females
• Diagnosis of hypertension	
• Uncontrolled hypertension or non adherence to prescribed medication or lifestyle changes after 12 months in QUICK - I	
• Age of 18 years and over	
• Give informed consent	

Healthcare Professionals for interviews on feasibility of CHEP:	
• HCP of OOH who participated in the implementation of CHEP, or	
• Managers and Doctors of HCHP	
• Give informed consent	

**Figure 1:**
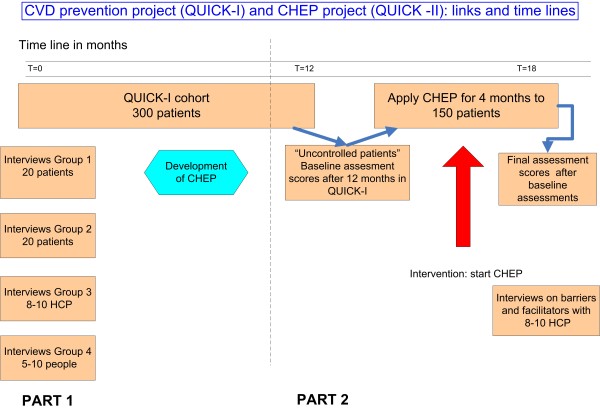
**Project populations and relationship between QUICK - I and CHEP study in QUICK - II**.

Figure 1 describes the project populations for QUICK - II and their relationship to the QUICK - I study.

### Outcomes

The primary outcome of the evaluation of CHEP will be the changes in adherence to medication or life style recommendations. This will be assessed through the Morisky self-report medication adherence questionnaire [14]. This scale asks patients to respond yes or no to five questions. Each positive answer is assigned a score of one, with higher scores indicating poorer adherence. The same scale will be used for other life style recommendations (e.g. salt intake). As such, self-reported adherence will be assessed as a continuous measure.

Secondary outcomes are changes in physiological measures - systolic blood pressure, diastolic blood pressure, and body mass index (BMI) or abdominal obesity at 6 months after baseline.

Table 2 indicates definitions of what we consider as significant improvement per outcome measure.

**Table 2:** Definitions of significant improvement per outcome measure

Outcomes	Significant improvement
Adherence to medication and life style recommendations (Primary outcomes)	Using the distribution on the Morisky scale [14] of low, medium, and high adherence ratios, a post CHEP effect shift of 10% to a higher category will be defined as a significant improvement in adherence.

Blood pressure (Secondary outcome)	Blood pressure decrease of >10% systolic or diastolic or blood pressure at target level (patients without diabetes or established CVD: <140 mmHg systolic and <90 mmHg diastolic, patients with diabetes, renal disease or established CVD: <130 mm Hg systolic and <80 mmHg diastolic).

Body mass index (kg/m^2 ^)	No CVD risk: <25 Moderate CVD risk: 25-30 High CVD risk: >30 In patients with a BMI >25 any reduction of BMI at 6 months will be regarded as an improvement.

Data will also be collected about other factors that may influence patients' hypertension management: self-reported cardiovascular risk factors (physical activity, diet, smoking, alcohol, sodium intake), knowledge of HTN and HTN management, perceptions of HTN, perceptions of medications, self efficacy, experienced stress, patient satisfaction with care (e.g. doctors' performance, supply of medication, frequency of follow up, satisfaction with CHEP etc).

### Sample size and Recruitment

#### Sample size calculation

The power analysis for sample size is based on the difference between the adherence score after implementation of CHEP and the adherence score at baseline using the 5-item Morisky adherence scale. Using the Morisky distribution of low, medium, and high adherence ratios of 32%, 52% and 16% respectively [14], and an estimated post CHEP effect shift of 10% to a higher category, we will need a minimum sample size of 150 patients.

#### Patient recruitment

The following steps will be applied in recruiting patients for the CHEP study:

1. Researchers will use the records of the QUICK - I study to identify all patients with uncontrolled hypertension or with low or medium adherence scores for medication or other life style measures on the Morisky Scale at 12 months after inclusion into the study.

2. Eligible patients will be informed about QUICK-II and invited for participation through a written invitation.

3. A research assistant will assess patients who are interested in participating for eligibility criteria and eligible patients will be asked to give informed consent.

4. Patients who give informed consent will be invited to a one-hour baseline assessment session.

5. Included patients who have completed baseline assessments will be invited to have three CHEP sessions spaced over a period of 4 months and one final assessment interview six months after baseline.

To encourage participation, all included patients will be reimbursed for extra travel costs incurred in visiting for assessments and CHEP sessions outside their normal clinic days.

### Intervention

The intervention (CHEP) will be developed in part 1 of this project. Subsequently, patients will be given (i) three CHEP sessions over a period of four months conducted by a trained nurse; (ii) audio-visual or written educational materials containing specific information for the target group and (iii) if necessary, referrals to regional facilities or initiatives that may help patients to adopt healthier lifestyles.

#### Sessions

The first session will take place two weeks after the baseline assessment interview, and the next two sessions will occur one and three months thereafter. Clinical guidelines generally recommend a patient centred approach as the preferred strategy for supporting patients in achieving CVD treatment goals, such as adherence to prescribed medication, dietary changes, and weight reduction, reduced sodium intake, increased physical activity and moderate use of alcohol [3]. While CHEP will use this framework, it will have the additional aim of eliciting and discussing culturally and socially specific aspects of patients' perceptions of cardiovascular risk factors and treatment. This method is based on the work of Arthur Kleinman [15], as well as more recent approaches to improving adherence in hypertensive patients of various ethnic and geographical background, such as those recently developed in The Netherlands [16]. In short, after identifying potential communication barriers and establishing a rapport with the patient, it is expected that the first session will focus on the patient's beliefs about hypertension. The next two sessions will deal with the daily challenges they face in achieving hypertension treatment goals within the broader context of their lives. Education will take place in group training sessions.

#### Educational materials

Patients will also be given information leaflets or audio visual materials that provide answers to frequently asked questions about hypertension. These will be designed to address the specific languages, customs, habits, norms and dietary cultures that characterize the communities of the patients participating in the program.

#### Supporting healthier lifestyles

If necessary, patients will be referred to initiatives offering healthier lifestyle support that is tailored to the target group, based on a referral list that will be established for this purpose in the first part of this study.

#### The nurses

In order to ensure treatment fidelity and to avoid organizational- and healthcare-related obstacles to implementation, the nurses who will provide CHEP will be given clear guidelines and extensive training in implementing these guidelines.

### Materials and Measurements

We will use the following measurements and materials to evaluate the effects of CHEP: patients' adherence to medication and other life style recommendations will be measured by using the five point Morisky Scale; and patients' blood pressure will be measured using pre validated OMRON M6 Comfort electronic equipment. The blood pressure will be measured on 3 occasions with the patient seated comfortably for 5 minutes, and the last two values averaged. All other physiological measures will be performed according standardized procedures using standard equipment. Other self-reporting measures (see Table 3) will be measured using the questionnaire of the OHD 2 trial in the Netherlands [16]. Aspects of this questionnaire will be adapted to the Nigerian specifics where necessary.

**Table 3:** Timelines and measures used in CHEP study

Measures	Baseline	Final
Physiological measures		
- Clinic BP measurements, Heart rate	X	X
- Height, weight, Body Mass Index	X	X
- Hip and waist circumference	X	X

Self-reporting measures		
- Patient demographics	X	
- Additional cardiovascular risk factors (physical activity, diet, smoking, alcohol, sodium intake)	X	X
- Medication adherence	X	X
- Adherence to lifestyle recommendations	X X	X X
- Knowledge of HTN	X	X
- Perceptions of HTN	X	X
- Perceptions of medication	X	X
- Self efficacy	X	X
- Satisfaction with care	X	X
- Perception of stress	X	X

Case file data		
- Prescribed medication	X	X
- Prescribed lifestyle measures	X	X
- Co-morbidity	X	X

Process data		
- Records office visits, patient drop-out data etc	X	X
- HCP interviews		X

All assessments will take place at baseline and 6 months after. A trained nurse will perform all physiological assessments. A trained interviewer will perform pre- and post CHEP assessment interviews. The researcher will be responsible for the training of the nurses and the interviewers.

Table 3 presents all the measurements that will be used in the study.

### Data Analysis

#### Statistical methods

The rate of adherence will be examined according to the intention to treat principle. Primary and secondary outcome measures will be calculated for every patient at baseline and 6 months thereafter to assess possible improvements. Furthermore, univariate and multi-level analyses will be performed to evaluate the modifying effect of the outcome measures (see above) on adherence or blood pressure. If the N is sufficient, we will perform separate subgroup analyses for gender. While a *p *value of 0.05 will be the critical value for all analysis, the *p *value will be adjusted for subgroup analysis according to standard procedures.

Data from interviews with HCP will be analyzed and used to identify potential barriers to the implementation of CHEP in practice.

## Ethical approval

Ethical approval was obtained for both QUICK studies [8].

## Discussion

Recent prevalence data from National Surveys indicate that many risk factors for CVD are highly prevalent in Nigeria: alcohol abuser/dependant - 4.4% (M - 8.1%, F - 0%); overweight/obesity (BMI ≥ 30 kg/m^2^) - 13.9% (M - 5.5%, F - 21.1%); physical inactivity - 6.8%; tobacco use - 9.9% (M - 19.3%, F - 1.8%); raised cholesterol - (M - 10.4%, F - 21.6%); raised blood pressure (systolic ≥ 160 mmHg or diastolic ≥ 95 mmHg) - 12.4% (M - 12.1%, F - 12.7%); and diabetes - 2.8% (M - 2.7%, F - 3%). There are no data on dietary intake such as fruits and vegetables http://www.who.int/research/en/. A substantial majority of CVD are preventable or treatable if patients at risk have access to quality CVD prevention and care programs. Quality care is, however, mostly inaccessible in resource-limited settings. Even where such care may be available and accessible, strict compliance and adherence to the dictates of therapy regimes becomes paramount for the successful control of CVD risk factors, and prevention of CVD in the affected populations. A recent study in Kwara state, Nigeria where QUICK is being implemented concluded that control of hypertension is unacceptably poor because of poor knowledge of hypertension and adverse practices by patients [17]. In a similar vein, another Nigerian study suggested that physicians should allocate special time for health education, having concluded that lack of time by physicians and inadequate knowledge about hypertension by patients are some of the potent barriers to effective CVD prevention and care [18]. Furthermore, with particular reference to SSA where deep routed cultural practices still play prominent roles in people's lives, health education to prevent CVD must be patient centered, and developed with due consideration for socio-cultural relevance in order for the intervention to be successful. By researching into the most optimal methods to implement an appropriately developed cardiovascular health education program, our expectation is that the present study will provide insight into, and contribute substantially to the improvement in CVD management and outcomes. We hope that the envisaged intervention, CHEP, will become productively applicable not only in Nigeria, but also in other similar settings in SSA and across the globe.

The study has one notable limitation:

The design of our evaluation of CHEP could have been improved by using a control group and a randomized design. However, the study is conducted in a very specific context, where it is impossible to avoid contamination. For example, patients have generally long waiting hours and may communicate about their condition or the treatment with each other. Moreover, the number of health care professionals in the clinic is limited such that it would be impossible to randomize them into a control and experimental condition. In addition, given the context of the community health insurance program, other clinics that provide standard cardiovascular care are difficult to find in the region. Nevertheless, to address this limitation, we consider analyzing the data collected during QUICK - I study for the patients included in QUICK - II to further compare and evaluate the effects of CHEP.

## Abbreviations

BMI: Body mass index; CHEP: Cardiovascular health education program; CVD: Cardiovascular diseases; DM: Diabetes mellitus; HCHP: Hygeia community health plan; HCP: Health care professionals; HIF: Health insurance fund; HMO: Health maintenance organization; HTN: Hypertension; ISH: International society of hypertension; OOH: Ogo oluwa hospital; QUICK: Quality improvement cardiovascular care Kwara; SSA: Sub Saharan Africa; WHO: World health organization.

## Competing interests

The authors declare that they have no competing interests.

## Authors' contributions

AOO drafted the manuscript, conducts the study and contributed to the design. MH, JH, CS, KS, and JL designed the study and they are members of the supervising board. AO, TA, SA, PA, KA were involved in the design of the study. JH revised several early drafts of the paper and KS, CS and MH commented on the final draft. All authors read and approved the final manuscript.

## Pre-publication history

The pre-publication history for this paper can be accessed here:

http://www.biomedcentral.com/1471-2458/11/171/prepub
